# Complexity of bacterial phosphorylation interaction network

**DOI:** 10.3389/fmicb.2014.00725

**Published:** 2014-12-17

**Authors:** Imrich Barák

**Affiliations:** Department of Microbial Genetics, Institute of Molecular Biology, Slovak Academy of SciencesBratislava, Slovakia

**Keywords:** *Bacillus subtilis*, protein phosphorylation, bacterial cell division, sporulation, BY-kinase

Protein phosphorylation is a vital mechanism in the regulation of all processes in eukaryotic and prokaryotic cells. It is one of the most important of those post-translational modifications which allow proteins to reversibly change their enzymatic activity, cellular localization, oligomeric state, half-life and interaction partners.

Protein phosphorylation is mainly used by bacteria to adapt to changes in their environment (where the conditions can alter rapidly), but it is also used for intercellular communication (reviewed in Kobir et al., 2011). An important part of the bacterial phosphorylation network is made up of two component systems, which are found only in bacteria and certain plants. The first component of these systems is a sensory kinase which autophosphorylates one of its histidine residues as a result of the recognition of a particular signal. This kinase then phosphorylates the second component, a response regulator, on an aspartate. Phosphorylated forms of the response regulator often bind specific DNA sequences at promoter regions, thereby regulating gene expression and thus triggering a cellular response. These systems are characterized by a high recognition fidelity between a given sensory kinase and its response regulator, with minimal cross-talk with other two-component systems. There does seem to be a wide range of additional regulators, however, which are able to interfere with the phosphotransfer reactions and thus join a given two-component system to other signaling pathways.

In bacteria, proteins can be phosphorylated on serine, threonine and tyrosine residues. Serine and threonine phosphorylation is mostly carried out by the Hanks family of serine/threonine kinases. Kinases from a different family, the protein-tyrosine kinases (BY-kinases) are normally used for tyrosine phosphorylation (reviewed in Chao et al., 2014). These BY-kinases do not have either sequence or structural homology with eukaryotic tyrosine kinases and they phosphorylate tyrosine residues using an ATP/GTP-binding Walker motif. BY-kinases take part in many different cellular processes, including DNA replication, sporulation, antibiotic resistance, heat shock response, biofilm formation and virulence.

In a recent paper in *Frontiers in Microbiology*, Shi et al. (2014a) described the protein-tyrosine phosphorylation network in *Bacillus subtilis*. Their wide interactome study identified many potential new substrates of kinases and phosphatases. Three findings in particular stand out: (i) their results clearly show that cross-talk does take place between the BY-kinase and the Hanks type Ser/Thr kinase interaction networks; (ii) new kinase substrates were found, including those involved in DNA replication, transcription regulation and cell division; and (iii) tyrosine kinases can be bound by several cytosolic or transmembrane modulators. One of the most interesting of these is MinD, a binding partner of kinase PtkA. They found that MinD modulates the kinase activity of PtkA *in vitro* and that MinD attracts PtkA to the cell poles. MinD is an ATPase which binds to the membrane as a dimer in its ATP-bound state through an amphiphatic helix. It attracts the cell division inhibitor MinC to the membrane and it also interacts with MinJ, which, in turn, interacts with DivIVA. DivIVA localizes to the negative curvature of the forming septum and persists at the cell poles together with the MinCDJ complex during vegetative growth. This mechanism appears to block asymmetric septation during vegetative growth. This blocking mechanism must be overridden before the first morphologically distinct stage of sporulation, the formation of a thin asymmetric septum, can occur.

It has been established that DivIVA has at least two different roles during sporulation. One role is to bind the DNA-binding RacA protein, thereby allowing proper chromosomal segregation to occur in the small part of the cell (the so-called forespore) after asymmetric cell division (Ben Yehuda et al., 2003). A second role, only recently described, is to localize the SpoIIE phosphatase to the site of asymmetric septation (Eswaramoorthy et al., 2014). On the other hand, there are no known roles for the MinD, MinC and MinJ proteins during sporulation (Barak, 2013). Although depleting any or all of these proteins has only a minimal effect on sporulation frequency (Cha and Stewart, 1997), it is still not possible to exclude the possibility that the Min system has at least a partial role in sporulation because a sporulation-like septum appears, in some *minD* mutant cells, to be misplaced from its normal polar site (Barak et al., 1998). In addition, quite different experiments have shown that MinCD-dependent repression of SpoIIIE assembly in the forespore is crucial for the proper segregation of the chromosome to the forespore after asymmetric septum formation (Sharp and Pogliano, 2002).

The study by Shi et al. (2014a) provides a large amount of additional data about interactions between and phosphorylation of the proteins involved in vegetative and asymmetric cell division and chromosome segregation during sporulation. In brief, they found new potential protein–protein interactions, new substrates and new modulators for kinases involved in the above mentioned processes. Interestingly, they showed that MinD is a modulator of the PtkA kinase activity and also serves as a determinant for its localization. Secondly they showed that DivIVA is a PtkA substrate and they found new putative interaction partners for SpoIIE. What would be the reason for such a wide interconnection between so many different phosphorylation networks? Perhaps the main reason is to finely tune different cellular processes and link them together (Shi et al., 2014b). In any case, all of this data gives a completely new twist to our former, more simplistic view of these processes; they show that they are in truth much more complex. Nevertheless, it is clear that many of their findings must still be shown to be biologically relevant, which will require additional work.

## Conflict of interest statement

The author declares that the research was conducted in the absence of any commercial or financial relationships that could be construed as a potential conflict of interest.
